# A Comparison between Conductive and Infrared Devices for Measuring Mean Skin Temperature at Rest, during Exercise in the Heat, and Recovery

**DOI:** 10.1371/journal.pone.0117907

**Published:** 2015-02-06

**Authors:** Aaron J. E. Bach, Ian B. Stewart, Alice E. Disher, Joseph T. Costello

**Affiliations:** School of Exercise and Nutrition Sciences and Institute of Health and Biomedical Innovation, Queensland University of Technology, Brisbane, Australia; Vanderbilt University, UNITED STATES

## Abstract

**Purpose:**

Skin temperature assessment has historically been undertaken with conductive devices affixed to the skin. With the development of technology, infrared devices are increasingly utilised in the measurement of skin temperature. Therefore, our purpose was to evaluate the agreement between four skin temperature devices at rest, during exercise in the heat, and recovery.

**Methods:**

Mean skin temperature (${\overset{-}{T}}_{sk}$) was assessed in thirty healthy males during 30 min rest (24.0 ± 1.2°C, 56 ± 8%), 30 min cycle in the heat (38.0 ± 0.5°C, 41 ± 2%), and 45 min recovery (24.0 ± 1.3°C, 56 ± 9%). ${\overset{-}{T}}_{sk}$ was assessed at four sites using two conductive devices (thermistors, iButtons) and two infrared devices (infrared thermometer, infrared camera).

**Results:**

Bland–Altman plots demonstrated mean bias ± limits of agreement between the thermistors and iButtons as follows (rest, exercise, recovery): -0.01 ± 0.04, 0.26 ± 0.85, -0.37 ± 0.98°C; thermistors and infrared thermometer: 0.34 ± 0.44, -0.44 ± 1.23, -1.04 ± 1.75°C; thermistors and infrared camera (rest, recovery): 0.83 ± 0.77, 1.88 ± 1.87°C. Pairwise comparisons of ${\overset{-}{T}}_{sk}$ found significant differences (*p* < 0.05) between thermistors and both infrared devices during resting conditions, and significant differences between the thermistors and all other devices tested during exercise in the heat and recovery.

**Conclusions:**

These results indicate poor agreement between conductive and infrared devices at rest, during exercise in the heat, and subsequent recovery. Infrared devices may not be suitable for monitoring ${\overset{-}{T}}_{sk}$ in the presence of, or following, metabolic and environmental induced heat stress.

## Introduction

Skin temperature assessment via contact (conductive) devices has historically been regarded as the primary measurement technique in clinical, exercise science and research settings [1, 2]. However, contact methods of skin temperature measurement present practitioners with a number of potential methodological limitations including: wire entanglement [3], loss of sensor contact during exercise [4], the formation of a microenvironment around the sensor due to the fixation method [5, 6] and relatively small spot measurements [7]. The advent of technology has seen non-contact infrared devices, without the associated conductive limitations, become widely accepted for skin temperature assessment [2, 8].

In exercise science and occupational settings, local skin temperature measurement allows for the estimation of heat strain [9], body mapping of thermoregulatory and perceptual responses [10], mean skin temperature (${\overset{-}{T}}_{sk}$) [11] and mean body temperature [12]. Furthermore, skin temperature measurement is essential in the calculation of skin-to-air heat transfer [13], dry heat loss [14] and evaporative heat loss capacity [15]. These measurements give insight into human thermophysiology while exposed to varying modalities of exercise [16], environmental extremes [17] and/or personal protective equipment [18].

Despite the widespread use of these devices throughout clinical, exercise science and research settings, empirical evidence supporting the agreement between infrared thermometry and conductive instruments is limited and equivocal [1, 4, 19–22]. Bilateral localised skin temperature differences within individuals have been commonly used to detect the presence of a variety of pathologies. The clinically significant threshold for healthy normalised skin temperature differences is 0.5°C [23, 24]. Consequently, mean differences between conductive and infrared devices measured at localised sites must not exceed this value [2, 20, 25, 26]. However, no recommendations pertaining to the classification of practically meaningful differences and limits of agreement between conductive and infrared devices for the measurement of ${\overset{-}{T}}_{sk}$ have been outlined.

There have been extensive investigations determining the validity of different conductive devices for the assessment of skin temperature using thermistors and iButtons [3, 27, 28]. To date, investigations comparing conductive and infrared devices during exercise are limited to only two small studies comparing devices during both rest and exercise [1, 4]; and only a single study measuring the effect of varying ambient temperatures on device accuracy [4]. Buono and colleagues [4] surmised that infrared thermometry is a valid means to measure ${\overset{-}{T}}_{sk}$ during rest and exercise in both hot and cold environments. These findings were based on a comparison between only two devices; thermistors and an infrared thermometer, and on a small sample of six female participants. In addition, ${\overset{-}{T}}_{sk}$ was not measured during post-exercise recovery when ${\overset{-}{T}}_{sk}$ decreases and returns to baseline. Employing a randomised crossover design Fernandes and colleagues [1], compared thermocouples and an infrared camera for monitoring ${\overset{-}{T}}_{sk}$ at rest, during exercise and subsequent recovery. Mean bias between the infrared camera and thermocouples were significant during rest (0.75°C), exercise (-1.22°C) and recovery (1.16°C); with the authors’ concluding that there was a poor correlation and low-reliability between the two measurement modalities. The devices were utilised separately in a distinct trial which was separated by at least two days. Although the authors attempted to control for the natural 24 hour circadian variation in skin temperature, by conducting testing at the same time of day, day-to-day variations in skin blood flow [29] and skin temperature [30] have previously been reported in healthy participants even when time of day is controlled for. Furthermore, a thermocouple was used as a reference measure and subsequently, conclusions made may be limited, as the accuracy and sensitivity (± 0.5°C and 0.1°C respectively) of the thermocouples is lower than that of most thermistors (e.g. ± 0.1°C and 0.01°C) [1].

This inconsistent evidence base [1, 4] has highlighted the requirement for a comprehensive investigation of conductive and infrared devices used to assess ${\overset{-}{T}}_{sk}$ in occupational, research, and exercise and sport science settings. To our knowledge, no study has simultaneously examined a range of devices commonly used to assess ${\overset{-}{T}}_{sk}$ during static (resting) and dynamic thermoregulatory responses as a result of environmental extremes (i.e. exercise in the heat and return to baseline). Further research is warranted that incorporates a larger sample and examines multiple devices simultaneously when participants are exposed to higher ambient temperatures, where device accuracy is of greatest importance. Therefore, the purpose of this study was to systematically evaluate the agreement between four commonly used skin temperature measurement devices—two conductive (thermistors and iButtons) and two infrared (infrared thermometer and infrared camera)—in the assessment of ${\overset{-}{T}}_{sk}$ during rest, exercise in the heat and subsequent post-exercise recovery.

## Materials and Methods

### Participants

After approval from the Queensland University of Technology’s Human Research Ethics Committee, 30 healthy, recreationally active males volunteered to participate in this study. The mean ± standard deviation (SD) values of the participants’ age, height, body mass, body mass index and sum of 8 skinfolds were 25.0 ± 2.9 yr, 1.82 ± 0.08 m, 78.7 ± 11.4 kg, 23.9 ± 2.2 kg·m^-2^ and 88.9 ± 32.3 mm, respectively. Prior to commencing the study all participants were given written information concerning the nature and purpose of the study, completed a health screen questionnaire and informed verbal and written consent was obtained in accordance with the Declaration of Helsinki. Contraindications for participation included a history, or current existence of any cardiopulmonary disease, acute skin conditions (e.g. adhesive tape allergy), any metabolic, arterial, venous or lymphatic pathology, current history of smoking, or the use of any medication that may alter cardiovascular function or thermoregulation.

### Experimental Protocol

Participants were instructed to avoid prolonged sun exposure five days prior to the testing day to prevent any sun burn. Where appropriate, any measurement site with exposed hair was shaved at least 36 hours before testing [31], to prevent inflammation or damage to the skin surface, which may artificially raise skin temperature and influence infrared measurements [32]. In preparation for testing, participants were instructed not to engage in exercise, ingest caffeine or alcohol 24 hours prior to testing [33], have a hot shower within two hours of arriving at the laboratory and to keep the measurement sites clean of ointments and cosmetics. All testing took place over a single session lasting approximately three hours in a sub-tropical locality during Australia’s Spring season (September, October) when the average outdoor conditions were 24.3 ± 2.5°C and 56 ± 10% relative humidity.

Data collection consisted of repeated skin temperature measurements taken by four commonly used instruments, over three sequential periods of rest, exercise in the heat and recovery. Acclimation, resting and recovery took place in a temperature-controlled, fluorescently lit room without the existence of electric heat generators, wind drafts or external radiation. Initially four body locations in accordance with ISO 9886 [11] were cleaned with alcohol swabs to remove any contaminants influencing skin emissivity [34] and marked for device allocation. The readings from these sites were used to calculate ${\overset{-}{T}}_{sk}$ using the following equation [11]:

$${\bar{T}}_{\text{sk}}=\left(T_{neck}\cdot 0.28\right)+\left(T_{scapula}\cdot 0.28\right)+\left(T_{hand}\cdot 0.16\right)+\left(T_{shin}\cdot 0.28\right)$$

Where *T*_*neck*_ represents the skin temperature (°C) of that region and the numerical value (e.g. 0.28) is the weighted value applied to that site. Participants wore training shoes, socks, underwear, and shorts for the duration of experimental testing.

Following a conventional 20 minute acclimation [35], resting measurements were taken during 30 minutes of seated rest in a air-conditioned environment (24.0 ± 1.2°C, 56 ± 8% relative humidity, <0.1 m·s^-1^ air speed). Participants were seated upright on an adjustable stool for the duration of the acclimation, resting and recovery periods. Participants were then moved inside of a controlled climate chamber (38.0 ± 0.5°C, 41 ± 2% relative humidity, 0.5 ± 0.1 m·s^-1^ air speed) to commence cycle ergometry (Ergomedic 824E, Monark, Vansbro, Sweden) performed at 120 watts for 30 minutes [36]. All subjects completed the 30 minutes of exercise in the heat. Following exercise, participants returned to seated rest in the air-conditioned laboratory environment (24.0 ± 1.3°C, 56 ± 9% relative humidity, <0.1 m·s^-1^ air speed) for an additional 45 minutes of data collection; in order to return to baseline ${\overset{-}{T}}_{sk}$. The required timeframe for participants ${\overset{-}{T}}_{sk}\;$to return to baseline was established through pilot testing.

### Skin Temperature Devices

Two conductive (EU-UU-VL5–0 Thermistors, Grant Instruments, Cambridge, UK; and DS1922L-F50 iButtons, Maxim Intergrated, Sunnyvale, California, USA) and two infrared (VisioFocus 06400 Infrared Thermometer, Tecnimed Inc., Varese, Italy; and A305sc Infared Camera, FLIR Systems, Wilsonville, Oregon, USA) measurement devices were used to assess skin temperature (specifications in Table 1). The thermistors, iButtons and the infrared thermometer were all new and unused, while the infrared camera had been recently serviced and internally calibrated by the manufacturer. Further to this, all devices were calibrated against a NIST-certified traceable thermometer (±0.06°C; TL1-W, ThermoProbe Inc., Pearl, Mississippi, USA) in a stirred water bath (350, Contherm Scientific Inc, Upper Hutt, New Zealand) between 22 and 42°C before human investigation [27]. The resultant calibration formula was applied to the recorded values following human data collection. The thermistor was identified as the comparative measurement criterion for this investigation due to its performance against the certified thermometer during calibration; mean bias = -0.02°C; 95% limits of agreement (LoA) = ± 0.06°C. Skin temperatures, air speed (Kestrel Pocket Weather 4000, Nielsen-Kellerman, Boothwyn, Pennsylvania, USA), ambient temperature and relative humidity (QuestTEMP 36, 3M, St. Paul, Minnesota, United States), were measured at three minute intervals during the rest (11 time points), exercise (11 time points) and recovery periods (15 time points). All skin temperature devices measured a given body site concurrently. A 50 mm by 50 mm square template was drawn on the skin at each of the four skin temperature sites marking the placement of the devices. Within the marked square, four 25 mm by 25 mm quadrants represented a measurement site for each of the four types of devices. In order to account for any within site variation, device allocation within each quadrant was determined with a random number generator (Research Randomizer Form v4.0, Social Psychology Network).

**Table 1:** Device Specifications.

Device:	Thermistor (Data Logger)	iButton	Infrared Thermometer	Infrared Camera
Model:	EU-UU-VL5–0 (SQ2020–2F8)	DS1922L-F50	Visiofocus 06400	A305sc
Make:	Grant Instruments^a^	Maxim Integrated^b^	Tecnimed^d^	FLIR Systems^c^
Range:	-50 to +150°C	-40 to +80°C	+1 to +55°C	-20 to +350°C
Sensitivity:	0.01°C	0.0625°C	0.1°C	< 0.05°C
Uncertainty:	± 0.1°C	± 0.5°C	± 0.3 at 20.0 to 35.9°C	± 2°C
			± 0.2 at 36.0 to 39.0°C	
			± 0.3 at 39.1 to 42.5°C	
			± 1.0 at 42.6 to 55.0°C	
IR Resolution:				320 × 240 pixels
Spectral Range:				7.5–14 μm

IR = Infrared. All specifications were derived from the corresponding manufacturer websites:

^a^ = http://www.grantinstruments.com/media/5118/temperature_and_humidity_probes.pdf

^b^ = http://datasheets.maximintegrated.com/en/ds/DS1922L-DS1922T.pdf

^c^ = http://support.flir.com/DsDownload/Assets/42901-1001_en_41.pdf

^d^ = http://www.visiofocus.com/specificheEN.html.

The four thermistors for each of the four body sites were connected to an associated data logger (Grant Instruments, Cambridge, UK) and laptop computer. The four iButtons were pre-programmed via the accompanying USB to computer receptor (DS9490R USB Port Adapter, DS1402D-DR8 Blue Dot Receptor, Maxim Integrated, Sunnyvale, California, USA) and set at an 11-bit resolution of 0.0625°C. Each conductive device was held in place on the participant’s skin with a single layer of tape (Premium White Leuko Sportstape, Beiersdorf, Hamburg, Germany) [5, 6].

The infrared thermometer was held at 90° from the skin’s surface with the aid of spacing rods that kept the device at a constant distance of 60 mm from the skin [37]. The infrared camera was positioned on a level tripod perpendicular to the seated, resting participant at a distance of 0.8–1.1 m depending on the height and size of the participant [33]. The camera was assembled and allowed to stabilise for at least 60 minutes prior to subject arrival [38]. Four 3 mm by 50 mm strips of aluminium tape (3M, St. Paul, Minnesota, United States) were used as inert markers and placed around the infrared camera quadrant to identify the region of interest during post-processing of the thermal images [39]. A constant skin emissivity for the infrared camera was set to 0.98 in accordance with previous research [40]. The infrared camera derived ${\overset{-}{T}}_{sk}$ from three thermal images that encompassed four regions of interest. Once fixed in place an infrared camera requires a stabilisation period of at least 30 minutes [38]; therefore, to maximise the number of between device comparisons the infrared camera remained in the air-conditioned laboratory to assess skin temperature before exercise and during recovery. As a result, ${\overset{-}{T}}_{sk}$ during exercise in the heat was recorded using the three remaining devices (thermistors, iButtons and infrared thermometer).

### Sample Size Calculation

An a priori power calculation revealed that a minimum of 18 participants were required to detect the smallest important change in means of 0.5°C between devices. This power calculation assumes a within-subject error of 0.5°C, and allows for a 5 and 20% chance of making a type I or type II error, respectively.

### Statistical Analysis

The absolute agreement between the thermistors and the tested devices were assessed by calculating the mean bias and 95% LoA. Mean bias is representative of the mean difference between thermistor and given device, while 95% LoA represents the probability the measurement difference is anticipated to range between. To account for the repeat measures on the same participant in the current study, modified LoA as proposed by Bland and Altman [41] were employed. A one-way analysis of variance (ANOVA) was performed to determine the within-participant variance and the between-participant variance; the following formula was used to then calculate a modified standard deviation:

$$ModifiedSD=\sqrt{\left(\left(MS_{B}-MS_{W}/m\right)+MS_{W}\right)}$$

where *MS*_*B*_ is the between-participant mean square, *MS*_*W*_ is the within-participant mean square and *m* is the number of repeated observations (when equal for all participants). Thereafter, the LoA were calculated as mean bias ± 1.96 modified SD.

For simplicity and practicality, a conservative mean bias greater than ± 0.5°C with LoA exceeding ±1.0°C from the thermistor would be practically meaningful and bring an instrument’s agreement into question. Data is presented as mean bias ± 95% LoA unless otherwise stated. All data were analysed using SPSS (SPSS version 21.0, SPSS Inc., Chicago, USA). Values of *p* < 0.05 were considered statistically significant.

Agreement and validity of each measurement instrument were tested with several measurement error statistical tests. Three separate, two-way repeated measures ANOVA (device * time) were used to analyse ${\overset{-}{T}}_{sk}$ at rest, during exercise in the heat and recovery. Normality was assessed using descriptive methods (skewness, kurtosis, outliers, and distribution plots) and inferential statistics (Shapiro—Wilk test). Where the assumption of sphericity was violated, the Greenhouse-Geisser (GG) epsilon was used to adjust the degrees of freedom to increase the critical values of the F-ratio.

To identify significant differences between the thermistors and the three other devices, paired sample t-tests post-hoc analysis, using a Bonferroni correction where appropriate, was performed across specific time points; at the beginning, middle and end of the resting period, and every second time point from the start of the exercise and recovery periods.

## Results

Mean bias ± 95% LoA during rest, exercise and recovery for the thermistors and iButtons were as follows: 0.01 ± 0.40°C, 0.26 ± 0.85°C, 0.37 ± 0.98°C; thermistors and infrared thermometer: 0.31 ± 0.44°C, -0.46 ± 1.23°C, 1.57 ± 1.75°C; thermistors and infrared camera (rest and recovery): 0.83 ± 0.77°C, 2.33 ± 1.87°C (Table 2).

**Table 2:** Agreement and validity of each tested device compared to the thermistor in each condition (n = 30).

Device	Analysis	Rest	Exercise	Recovery
iButton	Mean Bias	0.01	0.26	0.37
	Upper 95% LoA	0.41	1.11	1.35
	Lower 95% LoA	-0.38	-0.58	-0.69
	p-value*	1.000	< 0.001	< 0.001
Infrared Thermometer	Mean Bias	0.34	-0.44	1.04
	Upper 95% LoA	0.78	0.79	2.79
	Lower 95% LoA	-0.10	-1.67	-0.71
	p-value*	< 0.001	< 0.001	< 0.001
Infrared Camera	Mean Bias	0.83	NR	1.88
	Upper 95% LoA	1.60	NR	3.75
	Lower 95% LoA	0.06	NR	0.01
	p-value*	< 0.001	NR	< 0.001

Values in °C; SD = standard deviation; LoA = limits of agreement; NR = not recorded;

*p-values acquired from two way repeated measures ANOVA testing statistical significance between thermistor and devices.

**Fig 1:**
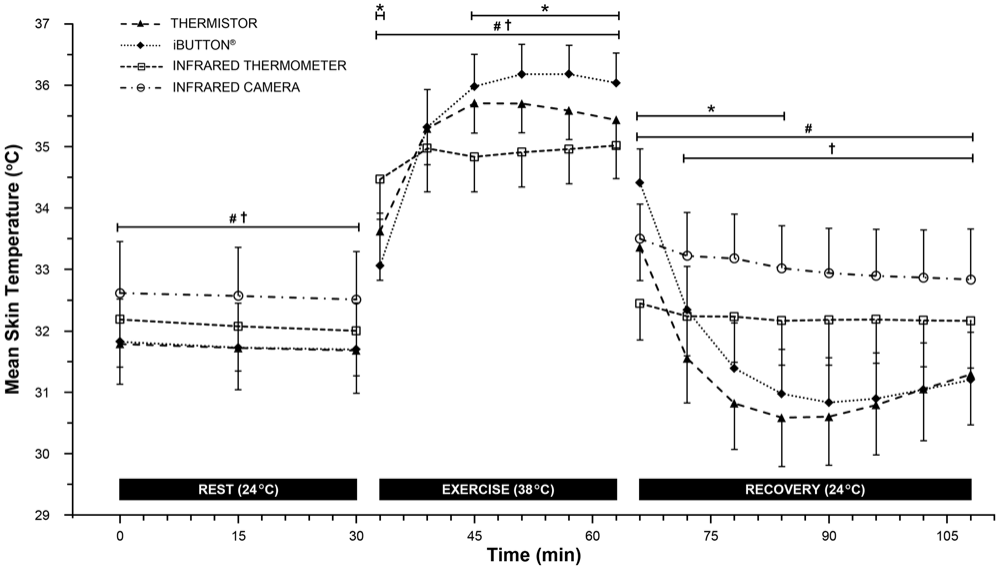
Mean skin temperature (*n* = 30) of all tested devices during rest (4 devices), exercise (3 devices) and recovery (4 devices). Post-hoc analysis displayed as *Significant differences between iButtons and thermistors (*p* < 0.001); ^#^Significant differences between infrared thermometer and thermistors (*p* < 0.001); ^†^Significant differences between infrared camera and thermistors (*p* < 0.001). Results are presented as the mean ± SD.

A significant main effects for device (rest: F_3,87_ = 103.77, *p* < 0.001 [Greenhouse-Geisser: GG], 1-β = 1.00; exercise: F_2,58_ = 154.08, *p* < 0.001 [GG], 1-β = 1.00; recovery: F_3,87_ = 178.87, *p* < 0.001, 1-β = 1.00), time (rest: F_3,87_ = 5.69, *p* = 0.008 [GG], 1-β = 0.8; exercise: F_2,58_ = 189.47, *p* < 0.001 [GG], 1-β = 1.00; recovery: F_10,290_ = 122.189, *p* < 0.001, 1-β = 1.00) and their interaction (rest: F_30,870_ = 2.37, *p* = 0.004 [GG], 1-β = 1.00; exercise: F_20,580_ = 155.54, *p* < 0.001 [GG], 1-β = 1.00; recovery: F_30,870_ = 125.08, *p* < 0.001, 1-β = 1.00) was observed in each of the three periods. Post-hoc analysis of individual time points for statistically significant differences between the thermistor and the other devices are indicated in Fig. 1.

## Discussion

This is the first study that has systematically evaluated the differences between multiple conductive and infrared devices frequently used to monitor skin temperature at rest, during exercise in the heat and recovery. This study provides evidence that conductive and infrared devices demonstrate poor agreement. Specifically, the key findings that emerged were: 1) significant systematic errors are present between conductive and infrared devices throughout rest, exercise in the heat and recovery, 2) infrared devices may not be sufficiently accurate to determine ${\overset{-}{T}}_{sk}$ during exercise in the heat and subsequent recovery, and 3) it should not be assumed that devices within acceptable agreement during resting conditions will continue to agree in the presence of environmental or metabolic changes. Our findings regarding the agreement between conductive and infrared devices contradict some previous investigations [2, 4, 19], although more recent evidence supports our results [1, 20, 22].

**Fig 2:**
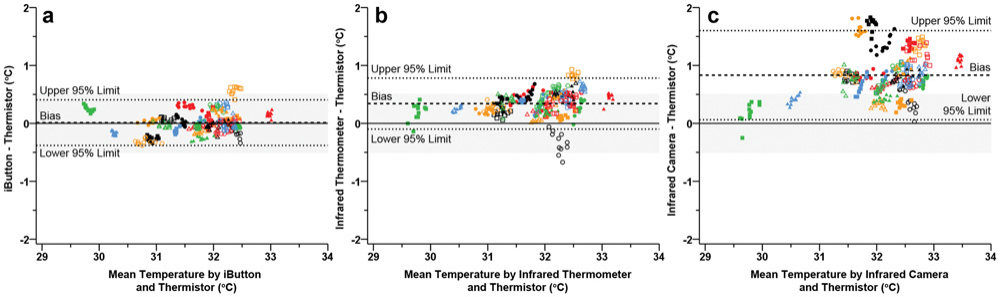
Bland—Altman plots during rest illustrating the mean bias (dashed line) and 95% limits of agreement (mean difference ± 1.96 · modified standard deviation: dotted lines) for thermistors vs iButtons (a), thermistors vs infrared thermometer (b) thermistors vs infrared camera (c). Each participant is represented by a unique colour or shape (*n* = 30). Grey band indicates the practically meaningful, a priori acceptable mean bias (± 0.5°C).

Across all time points measured during the rest period, the infrared camera significantly overestimated ${\overset{-}{T}}_{sk}$ compared to the thermistor, with a mean bias of 0.83 ± 0.77°C (Fig. 1). Significant differences precluding the agreement of an infrared camera and thermistor have previously been observed [1, 21]. However, the differences in the current study are not consistent with those of van den Heuvel and colleagues [21], where a previously calibrated infrared camera underestimated skin temperature by-2.32 ± 0.54°C when compared to a thermistor. These discrepancies could be attributed to the methodological differences, such as data collection and instrument models, between the studies. Specifically, van den Heuvel and colleagues [21] attached thermistors with a ‘minimal amount’ of collodion adhesive glue under the sensor head, and infrared camera analysis were conducted by selecting the skin ‘immediately adjacent’ to the thermistor tip. In combination with any subjective variance when using this method of post processing analysis, any excess adhesive dispersing outside of the thermistor tip could cause the infrared camera to measure the cooler temperature of the adhesive on the skin and not the skin itself. In a more recent study, Fernandes and colleagues [1] found an infrared camera overestimated ${\overset{-}{T}}_{sk}$ compared with thermocouples; 0.75 ± 0.78°C during rest in a laboratory environment (24.8°C, 61.9%), which is analogous to that of the current study; 0.83 ± 0.77°C. Nevertheless, the current body of research suggests that an infrared camera does not sufficiently agree with more traditional conductive methods of skin temperature measurement under stable resting conditions. In contrast, mean bias and 95% LoA (Fig. 2) were within the proposed practically meaningful limits for both the iButtons (0.01 ± 0.40°C) and the infrared thermometer (0.34 ± 0.44°C) under stable resting conditions. These findings suggest that the infrared thermometer presents as a viable low-cost alternative to that of relatively expensive infrared cameras as a means of accurate skin temperature measurement in static resting conditions; such as clinical diabetic ulceration prevention [42].

**Fig 3:**
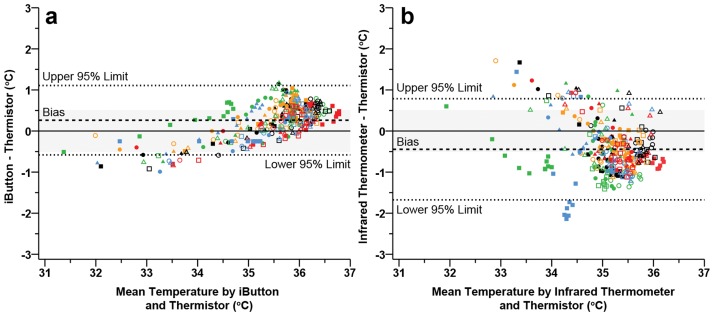
Bland—Altman plots during exercise in the heat illustrating the mean bias (dashed line) and 95% limits of agreement (mean difference ± 1.96 · modified standard deviation: dotted lines) for thermistors vs iButtons (a), thermistors vs infrared thermometer (b). Each participant is represented by a unique colour or shape (*n* = 30). Grey band indicates the practically meaningful, a priori acceptable mean bias (± 0.5°C).

During exercise in the heat, considerable differences and temperature fluctuations were observed between infrared and conductive measurement devices. Post-hoc analysis indicated significant differences between both the iButtons and the infrared thermometer, against the thermistors across exercise in the heat (Fig. 1). Initial increases in skin temperature while exercising in the heat are predominantly a function of ambient temperature and are not dependent upon workload per se [43]. Therefore, the initial differences observed between the three devices are most likely a result of individual thermal inertia of the two conductive devices and the infrared thermometers’ exposed skin site equilibrating to the change in ambient temperatures. The observed differences between the iButtons and thermistors at the start and towards the end of exercise are in line with previous observations [3, 27]. However, Bland—Altman plots examining the agreement between the two conductive methods identified that the acceptable limits were not violated (0.26 ± 0.85°C; Fig. 3). Therefore, the iButtons demonstrated sufficient agreement with the thermistors during exercise in the heat (Fig. 3) despite significant differences (Fig. 1). In regards to previous research, differences reported between iButtons and thermistors by Harper-Smith and colleagues [27], closely resemble our recent findings with a mean bias of 0.28°C.

Some evidence suggests that infrared thermometer sufficiently agrees with traditional thermistors for the measurement of skin temperature in the presence of environmental stressors [4, 20]. Our observations showed poor agreement (-0.44 ± 1.23°C; Fig. 3) where practically meaningful and statistical significant limits were not met during exercise in the heat. Although Buono and colleagues [4] monitored ${\overset{-}{T}}_{sk}$ via an infrared thermometer and thermistors during exercise in the heat, the authors calculated a correlation coefficient and not the agreement between instruments. A correlation coefficient is a measure of the strength of linear dependence between two variables and does not measure the strength of the agreement [44]. In order to ensure that two separate measurement techniques are classified as interchangeable (i.e. in agreement), more robust techniques such as the comparison of mean differences are needed [45]. Although not recorded in the current study, the amplified differences between the infrared thermometer and thermistors as exercise progressed may be a product of cooler sweat pooling and evaporating from the surface of the skin. Consequently, this would likely mean that the presence of sweat changes the radiant characteristics of the skin and influences ${\overset{-}{T}}_{sk}$ measurement from the infrared thermometer [34]. Moreover, the insulating characteristics of the tape covering both the thermistor and iButton would create an insulating microenvironment [5, 6], limit evaporative cooling, and therefore increase the temperature relative to the exposed infrared measurement site [46]. The impact of sweat pooling on the accuracy of recording ${\overset{-}{T}}_{sk}$ warrants future study.

The infrared camera exceeded the acceptable differences during rest and recovery. Despite not measuring ${\overset{-}{T}}_{sk}$ with the infrared camera during the exercise period, it is logical to suggest that the infrared camera would have significantly differed from that of thermistors. It is likely that the influence of sweat along the skin’s surface may see relative changes comparable to that of the infrared thermometer during the exercise. This assumption is supported by the recent findings of Fernandes and colleagues [1] whereby an infrared camera progressively underestimated ${\overset{-}{T}}_{sk}$ throughout exercise relative to the comparative thermocouples (1.22 ± 1.39°C). This effect has also been observed in thermal imaging cameras in which liquids present along the skin surface cause significant underestimation of true skin temperatures [34]. Moreover, even if relative, and not absolute, changes in ${\overset{-}{T}}_{sk}$ are of interest to researchers and exercise scientists, the use of infrared devices may not be suitable in settings where the participant is subjected to hot environments or exercise that induces a thermoreffector response.

**Fig 4:**
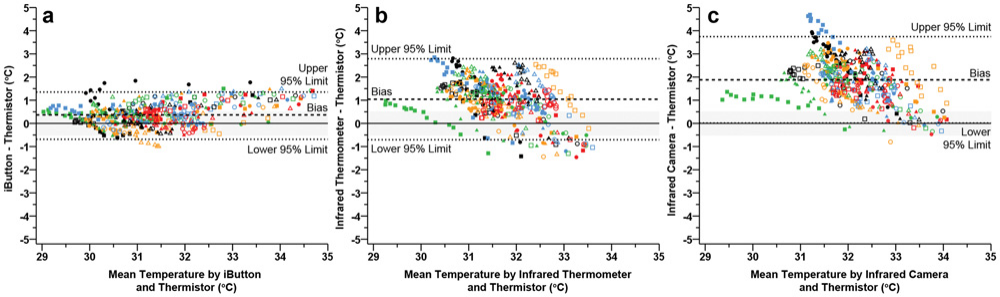
Bland—Altman plots during recovery illustrating the mean bias (dashed line) and 95% limits of agreement (mean difference ± 1.96 · modified standard deviation: dotted lines) for thermistors vs iButtons (a), thermistors vs infrared thermometer (b) thermistors vs infrared camera (c). Each participant is represented by a unique colour or shape (*n* = 30). Grey band indicates the practically meaningful, a priori acceptable mean bias (± 0.5°C).

To our knowledge, this is the first study that has compared infrared and conductive devices for the calculation of ${\overset{-}{T}}_{sk}$ during recovery from exercise in the heat. The present findings demonstrate systematic differences between conductive and infrared means of ${\overset{-}{T}}_{sk}$ measurement (Fig. 1 and 4). Of the devices tested, only the iButton was within the acceptable limits during recovery from exercise in the heat (0.37 ± 0.98°C). Both statistical and practically meaningful differences were present between the thermistor and both infrared instruments throughout the recovery period (infrared thermometer: 1.04 ± 1.75°C; infrared camera: 1.88 ± 1.87°C). We noted similar relative changes in skin temperature during exercise and recovery between conductive and infrared devices (Fig. 1) compared to Fernandes and colleagues [1]. Lower absolute mean differences (1.16 ± 1.32°C) reported by Fernandes and colleagues [1] may be a result of exposing participants to lower ambient temperatures during exercise (23°C). Findings from this study add to the current literature pertaining to the application of infrared devices in sports medicine and exercise science, by suggesting that conductive and infrared devices should not be used interchangeably during exercise in the heat or subsequent recovery. These findings also suggest that in a controlled laboratory environment, thermal imaging is not in agreement with more traditional hard wired conductive measures when assessing$\;{\overset{-}{T}}_{sk}$. Moreover, comparisons of thermoregulatory ${\overset{-}{T}}_{sk}$ responses between studies within the existing exercise science literature may not be feasible, if different means of cutaneous temperature measurement (conductive vs. infrared) were utilised during and/or following exercise in the heat.

Interpretation of our data is subject to certain limitations. First, due to the conductive devices being in direct contact with the skin, comparisons made between devices were measured from sites adjacent to each other and not a single point on the skin. Skin temperature variation within the measurement area could contribute to measurement differences between devices [47]. However, as there are no standardised anatomical placements for ${\overset{-}{T}}_{sk}$ devices within a region of interest, the uniform measurement area (50 mm^2^) and the randomised allocation of devices within the area, minimised errors associated with variation across the site. Secondly, it should be noted that the results are limited to the four most commonly used skin temperature devices. Future research should build upon the findings of the current study by assessing measurement differences between devices during resting and exercise conditions (of differing intensities and modalities) in a wider range of ambient temperatures. Finally, additional research is required to determine if the findings reported in this study are applicable to females, older participants, individuals with higher adiposity and the influence of sweat on both infrared and conductive devices.

In conclusion, this investigation showed poor agreement between conductive and infrared devices at rest, during exercise in the heat and subsequent recovery. More specifically, the infrared camera consistently overestimated ${\overset{-}{T}}_{sk}$ resulting in poor agreement and significant differences compared to the criterion thermistor. The results also indicate that iButtons and infrared thermometers are interchangeable with thermistors under stable resting conditions. However, agreement between the infrared thermometer and thermistors is not maintained during exercise in the heat or during subsequent recovery. These findings suggest that infrared devices are not suitable for monitoring ${\overset{-}{T}}_{sk}$ in the presence of, or following, metabolic and environmental induced heat stress.
